# Understanding skeletal muscle wasting in critically ill patients

**DOI:** 10.1186/s13054-014-0617-7

**Published:** 2014-12-03

**Authors:** Frank Lodeserto, Sachin Yende

**Affiliations:** Department of Critical Care Medicine, University of Pittsburgh, 606D Scaife Hall, 3550 Terrace Street, Pittsburgh, PA 15261 USA; The Clinical Research, Investigation, and Systems Modeling of Acute Illness (CRISMA) Center, University of Pittsburgh, 3550 Terrace Street, Pittsburgh, PA 15261 USA

## Expanded abstract

### Citation

Puthucheary ZA, Rawal J, McPhail M, Connolly B, Ratnayake G, Chan P, Hopkinson NS, Phadke R, Dew T, Sidhu PS, Velloso C, Seymour J, Agley CC, Selby A, Limb M, Edwards LM, Smith K, Rowlerson A, Rennie MJ, Moxham J, Harridge SD, Hart N, Montgomery HE: **Acute skeletal muscle wasting in critical illness.***JAMA* 2013, **310:**1591-1600.

### Background

Survivors of critical illness demonstrate skeletal muscle wasting with associated functional impairment.

### Methods

*Objective*: The objective was to perform a comprehensive prospective characterization of skeletal muscle wasting, defining the pathogenic roles of altered protein synthesis and breakdown.

*Design*: Prospective observational study.

*Setting*: Patients were recruited from a university and community hospital in England.

*Subjects*: The study involved 63 critically ill patients >18 years old who were anticipated to be intubated >48 hours and to spend >7 days in the ICU and to survive their ICU stay. Subjects were enrolled within 24 hours of admission.

*Outcomes*: Muscle loss was determined through serial ultrasound measurement of the rectus femoris cross-sectional area (CSA) on days 1, 3, 7, and 10. In a subset of patients, the fiber CSA area was quantified along with the ratio of protein to DNA on days 1 and 7. Histopathological analysis was performed. In addition, muscle protein synthesis, breakdown rates, and respective signaling pathways were characterized.

### Results

There were significant reductions in the rectus femoris CSA observed at day 10 (−17.7%; 95% confidence interval (CI), −25.9 to 8.1%; *P* <0.001). In the 28 patients assessed by all three measurement methods on days 1 and 7, the rectus femoris CSA decreased by 10.3% (95% CI, 6.1 to 14.5%), the fiber CSA by 17.5% (95% CI, 5.8 to 29.3%), and the ratio of protein to DNA by 29.5% (95% CI, 13.4 to 45.6%). Decrease in the rectus femoris CSA was greater in patients who experienced multiorgan failure by day 7 (−15.7%; 95% CI, −27.7 to 11.4%) compared with single organ failure (−3.0%; 95% CI, −5.3 to 2.1%; *P* <0.001), even by day 3 (−8.7%; 95% CI, −59.3 to 50.6% versus −1.8%; 95% CI, −12.3 to 10.5%, respectively; *P* =0.03). Myofiber necrosis occurred in 20 of 37 patients (54.1%). Protein synthesis measured by the muscle protein fractional synthetic rate was depressed in patients on day 1 (0.035%/hour; 95% CI, 0.023 to 0.047%/hour) compared with rates observed in fasted healthy controls (0.039%/hour; 95% CI, 0.029 to 0.048%/hour; *P* =0.57) and increased by day 7 (0.076%; 95% CI, 0.032 to 0.120%/hour; *P* =0.03) to rates associated with fed controls (0.065%/hour; 95% CI, 0.049 to 0.080%/hour; *P* =0.30), independent of nutritional load. Leg protein breakdown remained elevated throughout the study (8.5; 95% CI, 4.7 to 12.3 μmol phenylalanine/minute/ideal body weight × 100 to 10.6; 95% CI, 6.8 to 14.4 μmol phenylalanine/minute/ideal body weight × 100; *P* =0.40). The pattern of intracellular signaling supported increased breakdown (*n* =9, *r* = −0.83, *P* =0.005) and decreased synthesis (*n* =9, *r* = −0.69, *P* =0.04).

### Conclusions

Among these critically ill patients, muscle wasting occurred early and rapidly during the first week of critical illness and was more severe among those with multiorgan failure compared with single organ failure. These findings may provide insights into skeletal muscle wasting and critical illness.

## Commentary

Critical illness myopathy (CIM) and critical illness polyneuropathy (CIP) are common problems affecting nearly 50% of critically ill patients and are major contributors to increased morbidity and mortality [1-3]. There is significant overlap between the clinical presentation of CIP and CIM, including symmetrical weakness, decreased or even loss of deep tendon reflexes, and weaning difficulties. Both CIP and CIM often lead to a prolonged ICU and hospital course as well as a need for rehabilitation [2-4]. Many studies have grouped these processes together due to difficulties in clinically differentiating them, and they are commonly referred to as ICU-acquired weakness or critical illness neuromyopathy [2-5].

Although the clinical symptoms of CIP and CIM may share common features, and frequently coexist, several studies have explored mechanisms underlying each condition. CIM or skeletal muscle wasting is a complex problem mediated by multiple factors. Many mechanisms for CIM have been proposed, including elevated proteolysis, decreased protein synthesis, and sodium channel inactivation leading to membrane inexcitability and nitric oxide production [3]. Proposed risk factors leading to muscle wasting are numerous, including immobilization [2,3], systemic inflammation [2,3,5], hyperglycemia [2,3], and nutritional factors [2,3].

Puthucheary and colleagues conducted the present prospective observational study to understand the mechanisms underlying skeletal muscle wasting. They were specifically interested in examining the relationship between protein homeostasis (anabolism versus catabolism) and skeletal muscle wasting.

The investigators studied 63 critically ill patients and performed serial ultrasound-guided assessment of the cross-sectional area of rectus femoris muscle to study muscle changes. They also performed muscle biopsies, looking specifically at decreases in myofiber area and ratio of protein to DNA (loss of muscle mass) to examine histopathological changes. Finally, they tried to approach the problem from a biochemical/molecular level by measuring the rates of muscle protein synthesis and breakdown after infusion of amino acid and incorporation into the muscle tissue. The study was rigorous and required serial measurements at many levels to help uncover a complex multifactorial process. The major conclusions are that muscle wasting occurred early in critical illness, was more profound in multiorgan failure, and is a consequence of impaired protein synthesis leading to a net catabolic state.

The authors’ two major conclusions have important clinical implications, especially for the timing of muscle wasting and its development despite enteral nutrition.

The first major conclusion was that muscle wasting occurred early (first 10 days) and was more profound in multiorgan failure. These findings support previous reports that CIM and CIP occur early in critical illness [5]. Unfortunately, few interventions are available to prevent or treat CIM; for instance, avoidance of risk factors, such as hyperglycemia [6], and early mobilization and rehabilitation [7].

Puthucheary and colleagues’ second major conclusion is that the net catabolic state, resulting in skeletal muscle wasting, is a consequence of decreased muscle protein synthesis rather than increased protein breakdown. The authors demonstrated this by showing that protein synthesis was similar on day 1 of critical illness compared with fasted healthy subjects, and increased by day 7 and was comparable with well-fed healthy subjects. On day 1 the muscle protein synthetic rate was decreased compared with muscle protein breakdown, resulting in an overall net catabolic state – and the same relationship persisted on day 7. This finding is supported by earlier studies suggesting that muscle catabolism is a result of impaired synthesis rather than elevated breakdown [8].

Of importance, the majority of these patients (82%) who demonstrated impaired protein synthetic rates were being fed continuously by the enteral route. The authors noted an association between increased protein delivery and muscle wasting. While this association may seem counterintuitive, prior physiologic studies demonstrated that the use of continuous intravenous amino acid infusions led to an initial increase in protein synthesis followed by inhibition of protein synthesis over time [9,10]. Although continuous enteral feeding is the mainstay of critical care practice, the findings of this study and others raise questions about the optimal amount of protein delivered and the feeding regimens used (continuous versus intermittent).

## Recommendation

This study confirms that skeletal muscle weakness occurs early during critical illness. Skeletal muscle wasting is largely due to depressed protein synthesis. Various modifiable and nonmodifiable factors influence skeletal muscle wasting. Future studies should examine the role of various interventions, including different feeding regimens, to prevent muscle wasting in critically ill patients.

## Abbreviations

CI: Confidence interval
CIM: Critical illness myopathy
CIP: Critical illness polyneuropathy
CSA: Cross-sectional area
